# Piloting very early infant diagnosis of HIV in Lesotho: Acceptability and feasibility among mothers, health workers and laboratory personnel

**DOI:** 10.1371/journal.pone.0190874

**Published:** 2018-02-07

**Authors:** Michelle M. Gill, Lynne M. Mofenson, Mamakhetha Phalatse, Vincent Tukei, Laura Guay, Matsepeli Nchephe

**Affiliations:** 1 Elizabeth Glaser Pediatric AIDS Foundation, Project SOAR (Supporting Operational AIDS Research), Washington, D.C., United States of America; 2 Ministry of Health, Maseru, Lesotho; 3 Elizabeth Glaser Pediatric AIDS Foundation, Project SOAR (Supporting Operational AIDS Research), Maseru, Lesotho; 4 The George Washington University, Department of Epidemiology and Biostatistics, Milken Institute School of Public Health, Washington, D.C., United States of America; University of British Columbia, CANADA

## Abstract

**Introduction:**

Mortality associated with in-utero HIV infection rises rapidly within weeks after birth. Very early infant diagnosis of HIV (VEID)–testing within 2 weeks of birth–followed by immediate initiation of antiretroviral therapy has potential to avert mortality associated with in-utero transmission. However, our understanding of acceptability and feasibility of VEID is limited.

**Methods:**

VEID was piloted in an observational prospective cohort of HIV-positive pregnant women and their infants in 13 Lesotho health facilities. Between March-July 2016, semi-structured interviews were conducted with HIV-positive women attending 6-week or 14-week postnatal visits and health workers (HWs) in 8 study facilities in 3 districts as well as with district and central laboratory staff. Interview themes included acceptability of birth and subsequent HIV testing and early treatment, perceived VEID challenges, and HIV birth testing procedures and how well they were performed.

**Results:**

Interviews were conducted with 20 women, 18 HWs and 9 district/central laboratory staff. Nearly all mothers perceived knowing their child’s HIV status at birth positively. Mothers and HWs did not indicate that birth testing affected subsequent acceptance of infant HIV testing or clinic attendance. HWs and laboratory staff reported weak follow-up systems for mothers with home deliveries, and concern regarding the increased workload associated with additional testing requirements. All groups reported turnaround time delays for EID, and that sometimes results were never received.

**Conclusions:**

Women, HWs, and laboratory staff found VEID acceptable and were supportive of national implementation of birth testing. However, they identified challenges within the EID system that could be exacerbated by adding a test to the diagnostic algorithm, such as delays in receiving test results, suggesting VEID may not be feasible in certain settings. Policymakers will need to consider whether adding birth testing or strengthening the current clinic and laboratory system is the most appropriate course of action.

## Introduction

Perinatally HIV-infected infants face rapid disease progression and mortality as high as 68% by age 2 years without antiretroviral therapy (ART) [1–2]. Mortality rises in the first few weeks of life, with an early peak as high as 20–30% between ages 8–12 weeks [1, 3–4].

Early infant HIV diagnosis (EID) is critical, as early ART initiation between ages 6–12 weeks has been shown to dramatically reduce early infant mortality by 76% and decrease HIV progression by 75% [5]. More recently, a study of South African infants starting ART at a median age of 8.4 weeks showed that 62% had advanced HIV disease (CD4 <25% or World Health Organization [WHO] clinical stage 3 or 4) before starting ART, even at this young age [6]. Thus, even earlier diagnosis, potentially in the first 48 hours of life, and immediate ART initiation might be desirable. Several studies suggest that an increased proportion of perinatal infections will be *in utero* transmissions when mothers are receiving antepartum ART, and hence HIV can be detectable on day 1 of life in a significant percentage of infected infants [7–8].

The current WHO-recommended algorithm for EID includes initial viral testing of HIV-exposed infants at age 4–6 weeks. However, in 2014, among the 21 Global Plan countries in Africa, only half of HIV-exposed infants received an HIV test in the first 2 months of life [9]. Even in countries with higher EID coverage, such as South Africa, as many as 50% of tested infants are either not started on ART or are lost to follow-up, due to delays between testing and return of results [10].

Very early infant diagnosis (VEID) and immediate ART initiation can prevent decline in immunologic function and clinical deterioration, and ultimately decrease early infant HIV-related mortality. Mathematical modeling determined that 6 weeks may no longer be the optimal age to diagnose perinatal HIV infections, and recommended consideration of VEID at birth, followed by a test at 10 weeks. A 10-week test alone would identify more perinatal infections and save more life years compared to birth, 6 or 14 week-testing. Adding a birth test would identify 2,110 more infections and result in over 27,000 additional life years saved than the 10-week test alone. [11].

While birth virologic testing appears to be an attractive intervention to reduce early HIV-related mortality, questions remain about implementing this approach on a broad scale. This qualitative study aimed to evaluate the acceptability and feasibility of adding birth HIV testing to the routine testing algorithm for infants born to HIV-positive women. This study describes birth testing experiences and perspectives from health workers (HWs), laboratory personnel, and HIV-positive postpartum women enrolled in an observational cohort study to evaluate the effectiveness of PMTCT services. As part of the cohort study, birth DNA-PCR testing (testing within the first 2 weeks of life) was added to the standard EID testing algorithm at age 6 and 14 weeks in 13 facilities in 3 districts of Lesotho. Given the small number of facilities implementing birth testing in the pilot phase and that birth testing was intentioned to follow the same processes for blood collection, specimen preparation and transfer and results communication already established for other EID tests, there were minimal additional measures put in place to support its introduction.

## Materials and methods

### Pilot description

Birth testing was incorporated into the standard EID procedures for specimen and result transfer in study districts. Infant blood was collected on dried blood spot (DBS) filter paper by nurses, who were responsible for specimen preparation, packaging, and documentation. Results were transferred from Maternal and Child Health (MCH) clinics at health centers and hospitals to the district laboratory, where specimens were registered and compiled. District laboratories then shipped them to the Lesotho National Reference Laboratory (NRL). After testing at the NRL, result slips were typically shipped back to district laboratories and then in turn transported by motorcycle to health centers. Study nurses or lay counselors delivered results to caregivers.

### Study design

This qualitative study utilized semi-structured interviews with HWs, laboratory personnel, and HIV-positive postpartum women.

### Study setting

The 3 districts for the overall cohort study were selected by the Lesotho Ministry of Health (MOH) as focus districts for the integration of PMTCT and nutrition services. After excluding all low-volume health centers, and including all 5 hospitals and 4 high-volume health centers from the districts, 4 health centers were randomly selected from the remaining medium-volume facilities. (Data were based on 2012 antenatal care [ANC] attendance figures.) Facilities were supported by either the MOH or the Christian Health Association of Lesotho (CHAL). For this study on VEID, interviews were conducted with HIV-positive postnatal women and HWs in 8 facilities purposively selected from the 13 cohort facilities piloting birth testing in the country. The selection process ensured at least 1 hospital and 1 health center were included from each of the 3 districts and both MOH and CHAL-supported facilities were represented. Since district laboratory personnel were only based in hospital settings, these interviews were conducted in all 5 hospitals that were part of the 13 cohort facilities.

### Study participants

HIV-positive women were eligible for interviews if they were enrolled in the cohort study and were attending their 6-week or 14-week postpartum visit. Attempts were made to recruit all mothers of children who were diagnosed as HIV-infected at birth regardless of visit timing. Approximately 3 interviews per facility were targeted. Facility-based cohort study nurses referred eligible women attending their routine visits to interviewers when they were present at study facilities.

HWs were eligible for the study if they were working at a study facility and involved in VEID testing or pre/post-test counseling. As birth testing was being piloted as part of the cohort study, only study nurses, many of whom were embedded as clinic staff, and lay counselors, performed VEID-related tasks and thus met this criteria. Facility nurses not part of the cohort study were ineligible, as they were not involved in any aspect of VEID. All HWs meeting the criteria and present on the days interviews were conducted were invited to participate.

Laboratory staff were recruited for interviews if they were working in 1 of the 5 hospitals in the cohort or the NRL. Since laboratory staff at hospitals only collect specimens to send to the NRL, complete paperwork, and distribute results to facilities once received, only 1 interview was targeted per hospital. If multiple laboratory staff were present on the day of the interview, 1 potential participant was selected at random for recruitment. NRL personnel were recruited through their supervisor; 4 of the 6 staff responsible for performing the DNA-PCR tests were recruited for participation.

Interviews took place March—July 2016, approximately two years after VEID had been introduced as part of the cohort study in July 2014. Interviews were conducted by 4 study personnel trained in human subjects’ protections, the study protocol, and qualitative data collection methods. Interviews were recorded and lasted 30 minutes to one hour. Interviews with HWs and laboratory staff were conducted in English, while interviews with study women were conducted in Sesotho. Topics for all participants included acceptability of early testing and treatment as well as benefits and challenges of VEID. Other topics addressed in HW and laboratory staff interviews included procedures (e.g. testing, counseling, preparing specimens, returning results, ensuring treatment initiation) and how those may differ at birth versus other time points.

### Data analysis

Recordings were transcribed by the same interviewer who conducted the interview, with Sesotho recordings simultaneously translated into English. Subsets of recordings were reviewed against the transcripts by study staff fluent in both English and Sesotho. MAXQDA (V10) was used to analyze qualitative data. Data were coded according to a standardized codebook by the interviewers. A small subset of transcripts were first coded by multiple reviewers to develop a standardized approach among the team. Questionable segments of coded text were resolved among the coders or by the Principal Investigator. Data were analyzed according to predefined constructs of acceptability and feasibility. Data were reduced to matrices and then textual summaries.

### Ethical considerations

Ethical approvals were received from the Lesotho MOH Research and Ethics Committee (EC) and the George Washington University Institutional Review Board (IRB). All participants provided IRB/EC-approved verbal informed consent prior to data collection. Verbal informed consent procedures were used to minimize risk of a confidentiality breach as a signed consent form would have been the only record linking these participants’ names to their participation in the study.

## Results

### Characteristics of semi-structured interview participants

Interviews were conducted with 20 HIV-positive women who were attending their 6-week (n = 13) or 14-week (n = 7) postnatal visit. Five women interviewed at 6-weeks had not yet received infant birth testing results. All women interviewed at 14-weeks who had a birth test had received their child’s results; 1 did not receive a birth test for her child because the testing window was missed following a home delivery. All test results were HIV-negative.

Interviews were also conducted with 18 HWs responsible for administering birth testing or delivering birth test results. Of these, 14 were study nurses and 4 were counselors. Years of professional experience ranged from less than 1 year to 15 years, with a median of 3 years (interquartile range [IQR] 2–9). Five district and 4 central laboratory staff were interviewed; 5 were laboratory technicians and 4 were laboratory technologists.

### Acceptability

VEID acceptability was defined by the perceived benefits and challenges of early testing and treatment initiation, acceptability of treatment and subsequent infant testing after birth, and perspectives on national policy, including selective (“high risk”) versus universal testing. Quotes illustrating the acceptability thematic areas are presented in Table 1.

**Table 1:** Illustrative quotes by women and health workers on VEID acceptability.

Acceptability thematic area	Illustrative quote
VEID benefits	*… It’s like*, *most of the time you will find that you’re interested to know your child’s status at birth when you are HIV-positive*, *you would like to know immediately after birth your child’s status… and you will have a good feeling when your child gets tested because you will be able to know their HIV status… (Woman)*
	*I feel it is important for it to be done…those who do not know [their child’s status]… would say they will take care of the baby yet they might end up doing the wrong things*. *It is good*, *even though one as a human gets scared and emotional*, *it is important*. *Yes*! *(Woman)*
	*The benefits are that we will know the status of child and the child who needs to be initiated will be initiated as soon as possible*, *that will be the benefit and the child will live a better life and a healthy life rather than waiting for 6 weeks*, *not knowing that there may be a virus inside the child’s body*. *(Health worker*, *Health Center)*
Acceptance of treatment and subsequent infant HIV testing	*It does not matter if the child is infected at birth or at 6 weeks or even at 12 months*, *12 years*. *The reaction is still going to be the same*. *(Health worker*, *Hospital)*
	*It is because the first time she was tested the infection was not detected*, *so it might be visible when she is growing up*, *so it is important to keep testing her*. *(Woman)*
VEID challenges	*If I find that [results] are not available*, *but have to draw her blood again yet I still do not know her status*, *[it] stresses me*, *wondering what the condition might be*, *yes*! *(Woman)*
	*…when it’s beyond two months*, *we then develop a doubt whether we will ever get them*. *We call NRL and ask where the results are*. *Sometimes they say they have never received such*, *then I would not know what had happened*, *but we would have drawn them within two weeks or two months*.* *.* *. *(Health worker*, *Hospital)*
	*… But birth testing*, *they feel that their children are still very young to be punctured and for the blood to be drawn from them… Sometimes they feel that it is unnecessary*, *whereas some feel that it is necessary…*.*Some of them are frightened; there is still that mindset that there is a possibility that a child may get infected and that birth testing alleviates their fear*, *knowing that ‘at least I will know the results of birth [testing] as soon as possible rather than waiting for 6 weeks*.*’ (Health worker*, *Health Center)*
Perspectives on national policy recommendations	*…Because we always talk about the four prongs of HIV infection…If we say there could be HIV infection during labor*, *what are we doing about it*, *this is the perfect thing that we should do about it*, *we should test babies at birth*. *(Health worker*, *Health Center)*

#### VEID benefits

Respondents from all 3 groups agreed that birth testing was important. Nearly all women positively perceived having their child tested or knowing their child’s HIV status at birth. The primary benefit of VEID cited by women and HWs was earlier knowledge of children’s HIV status. Women felt this knowledge was important, for reasons such as being able to provide the appropriate care for the child and so that the child does not become ill later. Both HWs and laboratory staff highlighted earlier treatment initiation as a key benefit leading to reduced infant mortality. HWs also noted that earlier receipt of test results allowed them to alleviate caregiver uncertainty and guide proper care for the child. Birth testing refusal was rare; the main reasons were fear of hurting the newborn, long waiting time for blood draw, and fear that their child could be HIV-positive. While most women had no negative perceptions of birth testing, VEID acceptance was sometimes the result of extensive counseling and the existing, trusting relationship between study nurse and patient.

#### Acceptance of treatment and subsequent infant HIV testing

HWs reported that treatment acceptance was not felt to be different between mothers learning their child was infected at birth versus other time points. Counseling and individual personalities were more relevant to treatment acceptability for children. Women and HWs also did not indicate that birth testing affected subsequent infant HIV testing acceptance nor did it negatively influence women’s motivations to attend postnatal services. The few HWs who encountered women who refused birth testing indicated that those women still accepted subsequent infant tests.

#### VEID challenges

While women felt birth testing was important, they still expressed concerns about infant testing not specific to test timing, such as possible infant transmission. Two women also expressed discomfort with pricking their newborn to obtain blood. Women, HWs, and laboratory staff cited that turnaround time (TAT) for result receipt was a challenge for all DNA-PCR tests. HWs estimated TAT to receive results at the MCH clinic was 2 weeks to 3 months at both health centers and hospitals, with some results never received by health facilities. A woman raised the issue of her child being tested again at 6 weeks, though she had not yet received results from the birth test. While not all HWs perceived a significant increase in resource burden as part of the birth testing pilot, those who were counselors and all laboratory staff felt that a national roll-out of VEID would result in increased workloads and further delays in TAT. Laboratory staff suggested that additional laboratory technologists, or data clerks to assist with sample registration and packaging, could help address the workload challenge at laboratories.

#### Perspectives on national policy recommendations

Despite challenges, HWs felt that birth testing was important and the majority recommended nationwide implementation. Most HWs and laboratory staff preferred universal birth testing for HIV-exposed children, rather than selective ‘high risk’ testing. They reasoned that all HIV-positive women should be considered high risk and assessing risk in clinic settings would be difficult. While in favor of broader implementation, one laboratory respondent suggested that VEID rollout be staggered, starting with hospitals and then to lower-level facilities, to better understand the extent of the added burden to the laboratory/health system and plan accordingly.

### Feasibility

VEID feasibility was defined as the procedures used for the HIV birth testing pilot and how well they were performed and could be practicably implemented on a national scale, including counselling on and understanding of birth testing, birth testing following home deliveries, communicating test results, and perceived challenges with VEID scale-up. Quotes illustrating the feasibility thematic areas are presented in Table 2.

**Table 2:** Illustrative quotes by women, health workers and laboratory personnel on VEID feasibility.

Feasibility thematic area	Illustrative quote
Birth test counselling and understanding	*I think it was because even before she said anything*, *I told her that before I left home for testing I had already counseled myself*. *I told myself that this is my life and I have to do the right thing to live and in the case of my baby as well*, *I knew that I was supposed to do the right thing*. *(Woman)*
	*…labor comes with emotions as women suffer many illnesses*, *post-natal depression or anything so at that time we have to understand that it might not mean she actually [refuses] but she might need some rest [after delivery]*. *So what I do is that I explain thoroughly to mothers ‘you know this is what needs to happen*, *the positives and the negatives…but I understand if you need time for me to explain further and after explaining*, *they gave me their go-ahead and not because I forced them*, *[but] because I still reassure them that ‘I respect your opinion*, *it is you who will give me the go ahead and if you say no*, *I cannot do it*.*’ (Health worker*, *Health Center)*
Birth testing following home delivery	*[Do they come here when they are 6 weeks only or 7 days*?*] …sometimes they complain the place is too far*, *no mode of transport*, *they struggle truly*. *You may find that a woman after delivering at 7 days*, *they are not yet fit to travel such long distance*, *it is truly very far*. *(Health worker*, *Hospital)*
Test result communication	*Everyone in MCH who sees [test] results that are positive*, *it’s their responsibility to call that lady*. *(Health worker*, *Hospital)*
	*I think if the results are positive we find ways of contacting the mother but if it’s negative she will be told when coming to the next visit at the clinic…when it’s positive*, *if we have the [phone] number we call*. *We also have people in the communities who track such people immediately*. *Such people are brought to the facility so that the infants would be initiated*. *(Health worker*, *Hospital)*
VEID scale-up challenges	*There are going to be more samples and that is going to affect turn-around time…because I think at national level there is going to be high workload and it means processing a lot of samples- we are going to get the results later than we get them now*. *(District lab personnel)*
	*This birth DNA-PCR*, *if it were a national thing*, *it will mean when we do health education*, *we would include it and we would also mobilize in the community…It would mean these mothers would know to come for a 7-day visit and would also mean that mothers would know that their children are tested at birth*. *(Health worker*, *Hospital)*

#### Birth test counselling and understanding

Most women understood the purpose of birth testing, felt that HWs had answered their questions, and first learned about birth testing in ANC. However, a significant number described the time spent in counseling as too short. Among these women, the amount of time being counseled varied from 5 to 35 minutes in ANC and at delivery combined. The majority of HWs estimated that counseling lasted 15–30 minutes, depending on women’s level of understanding and questions. HWs reported that women’s questions included whether the child was too young to have blood drawn or was being hurt unnecessarily, what to do if the child’s result was positive, and when results could be expected.

#### Birth testing following home delivery

Home deliveries were cited as a significant challenge to universal birth testing, particularly in more remote facilities. Women who deliver at home or in South Africa may not return to the facility within the 2-week window to have their infants tested. In order to help ensure a birth test was conducted, village or community health workers would accompany women and their newborns to a facility if possible or report home deliveries to the MCH clinic for follow-up. Other strategies employed by HWs included asking women to notify them in case of home delivery, and following up with women via phone around the time of their estimated delivery date.

#### Test result communication

Nearly all HWs described using mechanisms to fast-track HIV-positive results to mothers or other caregivers. Typically, they called mothers on the same day the result was returned and enlisted community or village health workers to locate them and facilitate their return to the facility if needed. This presented challenges when women lived outside the surrounding villages and when there was poor mobile network coverage or unavailable phone numbers. HWs described waiting to share HIV-negative infant results until women attended their regularly scheduled clinic visits, occurring every 1 to 2 months. Women also described receiving their child’s results at scheduled visits.

No formal fast-tracking mechanisms for HIV-positive results existed at the laboratory level, however some laboratory staff reported hand-delivering results to clinics (for those on the same campus) or calling health centers if there was a positive result. The reason given for results not typically being relayed by phone was so that the results can be properly documented in patients’ health booklets.

#### VEID scale-up challenges

While nearly all respondents from the 3 groups felt birth testing should be rolled out nationally, they anticipated challenges moving from pilot-testing to national implementation. HWs and laboratory staff described even longer TAT for results with an additional burden on staff. Laboratory respondents were concerned that having samples collected at any time for deliveries versus having scheduled days for blood draws would affect transportation schedules at health centers. Other challenges included the limited capacity of machines to perform additional tests and the greater likelihood of reagent stock-outs at central level. HWs also described issues that were reflective of the pilot nature of birth testing, such as the lack of birth testing information in standard facility registers and group counseling messages that omitted birth testing and failed to emphasize the 7-day visit for infants not tested at birth. They felt these issues would be addressed if the algorithm is updated in the national guidelines.

Given the delay of transporting samples and results, several HWs and laboratory respondents suggested DNA-PCR machines be placed in districts to bring EID services closer to women. Other recommendations to reduce TAT included improving communication from the district and central laboratories to MCH clinics, so positive test results could be immediately communicated and documentation errors could be swiftly resolved, and ensuring the laboratory information system and short message service (SMS) printers are functional to expedite result transfer. It was also suggested to communicate HIV-negative results to women via text messaging and include prevention strategies for keeping their child negative.

## Discussion

Our study provides some of the first published qualitative findings on acceptability of birth testing. We found that birth testing was acceptable to women, HWs, and laboratory staff and that there was support for national implementation of birth testing. However, they identified EID system challenges that could be exacerbated by adding a test to the diagnostic algorithm, suggesting VEID may not be feasible in certain settings, unless systems were strengthened. Respondents felt that adding birth testing to the current EID specimen and result transfer system would likely increase workloads and result in further delays in TAT. In addition, ensuring HIV-exposed infants delivered at home were tested soon after birth would be a challenge due to weak follow-up systems for tracing HIV-exposed infants and limited attendance at 7-day postnatal visits.

Interviewed women regarded knowing their child’s status around the time of delivery positively. Birth test refusals were rare, similar to another study reporting 100% uptake of birth testing [12]. HIV-positive women in Kenya, Namibia and Nigeria who had not undergone birth testing saw the benefit in knowing the child’s status earlier, but noted potential concerns if VEID were implemented, including discouraging subsequent testing if birth test was viewed as definitive [13]. Participants in our study did not indicate that VEID would affect subsequent postnatal attendance for EID testing. While interim quantitative data collected from this cohort demonstrated that nearly all women whose infants had a birth test performed and were eligible for a 6-week test returned for this test, having a birth HIV test in South Africa decreased the likelihood that infants would receive the 6-week DNA-PCR test on the standard schedule [14–16].

Improvements along the EID pathway would be critical to a successful VEID rollout. Decentralization of testing to the district level and use of point-of-care (POC) HIV diagnostic machines could permit more timely treatment initiation for infants identified as HIV-positive. It would help to address some challenges related to transportation of samples and results, laboratory staff shortages by task shifting of testing, and the limited capacity of a small number of machines at central level. However, this strategy would need to be evaluated for cost-effectiveness and feasibility of “hub and spoke” models. While POC diagnostics has demonstrated promise for other tests, such as CD4, it is not without challenges, including increased provider workload [17,18].

Strengthening clinic and laboratory systems to improve EID at 6 weeks of life rather than the addition of birth testing should also be considered. A study in South Africa suggests that, under certain conditions, focusing on DNA-PCR testing at 6 weeks would be more effective. A 6-week plus birth testing scenario with 50% testing coverage and 50% of results returned, yielded a lower infant survival gain than if 6-week testing coverage and result return frequency was increased to 75% for 6-week testing alone [19]. In countries like Lesotho with high coverage of virologic testing by 2 months of age (93%), an improvement in test result receipt at 6 weeks is a viable option to improve outcomes for HIV-positive infants [20]. While early ART initiation in infected infants has been shown to reduce morbidity and mortality, if results are not returned when the mother-infant pair returns for their 6-week visit, the benefits of early testing are diminished.

If birth testing were to be implemented nationally, follow-up systems for women delivering at home would need to be strengthened. Counselling messages would need to emphasize the importance of birth testing and the 7-day visit. Given that the Lesotho Demographic and Health Survey found that nearly a quarter of women delivered at home [21], the potential for missing the birth test window is considerable.

While respondents favored universal testing, a targeted testing program of infants at high risk of *in utero* infection might be a cost-effective way to implement birth testing, particularly when risk of MTCT is low. However, a targeted program may be more complex to implement, and could result in high-risk infants not being identified and hence missing birth testing. A study on birth testing in South Africa estimated that targeted testing, based on risk factors such as short maternal ART duration, poor adherence, and infant low birth weight, would miss 36% of infected newborns [13].

The study had limitations. Due to low numbers of HIV-infected children during the study period, no women who had a child infected at birth were interviewed, despite efforts to include them. No interviews were conducted with clinic (non-study) nurses. These nurses were not involved in VEID as it was only a pilot, though they may have viewed VEID less favorably. While they would certainly be involved if VEID were rolled out nationally, counselors, who were interviewed as part of this study, would be primarily responsible for performing infant HIV counseling and testing. Finally, given the qualitative nature of the study and the limited perspectives permitted by the pilot, we are not positioned to make a recommendation regarding widespread implementation. However, this is one of the first qualitative studies on VEID and offers some lessons learned for how to optimize success should birth testing be rolled out nationally.
